# Endovascular Therapy for Stroke Presenting Beyond 24 Hours: A Systematic Review and Meta-analysis

**DOI:** 10.1001/jamanetworkopen.2023.11768

**Published:** 2023-05-04

**Authors:** Hassan Kobeissi, Sherief Ghozy, Gautam Adusumilli, Ramanathan Kadirvel, Waleed Brinjikji, Alejandro A. Rabinstein, David F. Kallmes

**Affiliations:** 1 Department of Radiology, Mayo Clinic, Rochester, Minnesota; 2 College of Medicine, Central Michigan University, Mount Pleasant; 3 Department of Radiology, Massachusetts General Hospital, Boston; 4 Department of Neurologic Surgery, Mayo Clinic, Rochester, Minnesota; 5 Department of Neurology, Mayo Clinic, Rochester, Minnesota

## Abstract

**Importance:**

Previous randomized clinical trials (RCTs) have supported the use of endovascular therapy (EVT) in late-window acute ischemic stroke (AIS; 6-24 hours). However, little is known about the use of EVT in very late-window AIS (>24 hours).

**Objective:**

To examine outcomes following EVT for very late-window AIS.

**Data Sources:**

A systematic review of the English language literature was conducted using Web of Science, Embase, Scopus, and PubMed to search for articles published from database inception until December 13, 2022.

**Study Selection:**

This systematic review and meta-analysis included published studies regarding very late-window AIS treated with EVT. Multiple reviewers screened studies, and an extensive manual search of the references of included articles was performed to identify any missed articles. Of the 1754 initially retrieved studies, 7 published between 2018 and 2023 were ultimately included.

**Data Extraction and Synthesis:**

Data were extracted independently by multiple authors and evaluated for consensus. Data were pooled using a random-effects model. This study is reported per the Preferred Reporting Items for Systematic Reviews and Meta-Analyses 2020 guideline, and the protocol was prospectively registered with PROSPERO.

**Main Outcomes and Measures:**

The primary outcome of interest was functional independence, as assessed with 90-day modified Rankin Scale (mRS) scores (0-2). Secondary outcomes included thrombolysis in cerebral infarction (TICI) scores (2b-3 or 3), symptomatic intracranial hemorrhage (sICH), 90-day mortality, early neurological improvement (ENI), and early neurological deterioration (END). Frequencies and means were pooled with the corresponding 95% CIs.

**Results:**

This review included 7 studies involving a total of 569 patients. The mean baseline National Institutes of Health Stroke Scale score was 13.6 (95% CI, 11.9-15.5), and the mean Alberta Stroke Program Early CT Score was 7.9 (95% CI, 7.2-8.7). The mean time from last known well and/or onset to puncture was 46.2 hours (95% CI, 32.4-65.9 hours). Frequencies for the primary and secondary outcomes were 32.0% (95% CI, 24.7%-40.2%) for functional independence (90-day mRS scores of 0-2) and 81.9% (95% CI, 78.5%-84.9%) for TICI scores of 2b to 3, 45.3% (95% CI, 36.6%-54.4%) for TICI scores of 3, 6.8% (95% CI, 4.3%-10.7%) for sICH, and 27.2% (95% CI, 22.9%-31.9%) for 90-day mortality. In addition, frequencies were 36.9% (95% CI, 26.4%-48.9%) for ENI and 14.3% (95% CI, 7.1%-26.7%) for END.

**Conclusions and Relevance:**

In this review, EVT for very late-window AIS was associated with favorable frequencies of 90-day mRS scores of 0 to 2 and TICI scores of 2b to 3 and with low frequencies of 90-day mortality and sICH. These results suggest that EVT may be safe and associated with improved outcomes for very late-window AIS, although RCTs and prospective, comparative studies are needed to determine which patients may benefit from very late intervention.

## Key Points

**Question:**

Is endovascular therapy (EVT) for acute ischemic stroke associated with favorable outcome distributions in the very late window (>24 hours)?

**Findings:**

In this systematic review and meta-analysis of 7 studies comprising 569 patients with a mean onset to puncture time of 46 hours, EVT in the very late window was associated with favorable frequencies of clinical and procedural outcomes. Furthermore, EVT was associated with a promising safety profile, with low frequencies of 90-day mortality and symptomatic intracranial hemorrhage.

**Meaning:**

These findings suggest that EVT may be safe and associated with favorable outcome distributions for patients with very late-window acute ischemic stroke, although future randomized clinical trials and prospective, comparative studies are needed to determine which patients may benefit from EVT beyond 24 hours.

## Teaser

This systematic review and meta-analysis examines outcomes after endovascular therapy for acute ischemic stroke presenting after 24 hours.

## Introduction

 Since the publication of several landmark randomized clinical trials (RCTs) in 2015 and a subsequent patient-level meta-analysis in 2016, endovascular therapy (EVT) has become the standard of care for eligible patients with acute ischemic stroke (AIS) due to large vessel occlusion (LVO).^1^ Early RCTs mostly tested EVT within 6 hours after stroke onset.

Two RCTs (DEFUSE-3^2^ and DAWN^3^) published in 2018 and a subsequent patient-level meta-analysis published in 2022^4^ reported that EVT was superior to medical therapy in AIS due to anterior circulation (AC)-LVO presenting in the late window. Additionally, patients presenting between 12 and 24 hours after last known well benefited from EVT more than those presenting between 6 and 12 hours.^4^ As such, EVT was deemed to be the standard of care for eligible patients presenting between 6 and 24 hours after last known well.

The efficacy and safety of EVT beyond 24 hours after last known well (the very late window) remains unknown. To our knowledge, no RCTs have been conducted in this patient population, although smaller studies have been published reporting outcomes following EVT in the very late window.^5,6,7,8,9,10,11^ Therefore, we conducted a systematic review and meta-analysis of published studies evaluating outcome distributions of EVT performed more than 24 hours after last known well.

## Methods

### Search Strategy

This systematic review and meta-analysis followed the *Cochrane Handbook for Systematic Reviews of Interventions*^12^ and the Preferred Reporting Items for Systematic Reviews and Meta-analyses (PRISMA) 2020 reporting guideline. The study protocol was prospectively registered with PROSPERO (CRD42023386868). In accordance with the Code of Federal Regulations, 45 CFR 46.102, this study was exempt from institutional review board approval and informed patient consent requirements were waived because this is a secondary use of deidentified data sets.

The systematic review was conducted with AutoLit software, version 1.46 (Nested Knowledge), using the PubMed, Embase, Web of Science, and Scopus databases, which were searched from inception to December 13, 2022. Based on each database, different combinations of possible keywords and/or Medical Subject Headings terms were used. Keywords and Medical Subject Headings terms included *stroke*, *thrombectomy*, *late, very late*, and others. The full search strategy is provided in the eAppendix in Supplement 1. Moreover, we performed an extensive manual search of the references of the included articles to retrieve any missed articles. One study^11^ was published following the initial search and was subsequently added manually on January 3, 2023.

### Screening Process and Eligibility Criteria

We included all original studies fulfilling our population (very late-window AIS [>24 hours]), exposure (EVT), comparator (none), and outcomes criteria. The primary outcome of interest was functional independence, assessed with 90-day modified Rankin scale (mRS) scores (0-2). Secondary outcomes of interest were thrombolysis in cerebral infarction (TICI) scores (2b-3 or 3), symptomatic intracerebral hemorrhage (sICH), 90-day mortality, early neurological improvement (ENI), and early neurological deterioration (END).

We excluded studies in which very late-window AIS was not reported, review articles, duplicate studies including the same patients presented in another included study, case reports, case series with fewer than 10 patients, and abstracts. We did not pose any limitations on patient characteristics or stroke location. Two authors (H.K. and G.A.) performed the title and abstract screening, which was followed by a full-text screening stage composed of studies remaining from the initial screening. In both stages, a third, senior author (D.F.K.) was consulted to reach consensus.

### Data Extraction

Data extraction was also performed with AutoLit software. The extracted data included study characteristics, baseline data of included patients, and outcomes of interest. Two authors (H.K. and G.A.) independently completed the extraction, which was subsequently evaluated for consensus by a third author (S.G.).

### Risk-of-Bias Assessment

The Newcastle Ottawa Scale^13^ was used to assess the risk of bias, and 2 independent reviewers (H.K. and G.A.) evaluated included studies. Study quality was rated as good (3 or 4 stars, 1 or 2 stars, and 2 or 3 stars in the selection domain, comparability domain, and outcome or exposure domain, respectively), fair (2 stars, 1 or 2 stars, and 2 or 3 stars in the selection domain, comparability domain, and outcome or exposure domain, respectively), or poor (0 or 1 star, 0 stars, and 0 or 1 star in the selection domain, comparability domain, and outcome or exposure domain, respectively).

### Statistical Analysis

We pooled frequencies and means with the corresponding 95% CIs. Generalized linear mixed models with logit transformation and random effects were used to pool data due to relatively small sample sizes, the presence of 0 events, and methodological differences between studies.^12,14^ Heterogeneity was assessed using the *Q* statistic and the *I*^2^ test, where *P* < .05 (2-tailed) or *I*^2^ values greater than 50% were considered significant.^15^ Funnel plots were used to visualize publication bias.^12,16^ All data were analyzed using R, version 4.2.2 (R Project for Statistical Computing), and the meta package.

## Results

### Search and Screening Results

After we removed 1081 duplicate records, we retrieved 1754 articles for further screening. We excluded 1742 records through the title and abstract screening stage to retain 12 records for full-text screening. Two records were identified via expert recommendation. Of these 14 studies, 7 were excluded (eFigure 1 in Supplement 1). Thus, 7 studies^5,6,7,8,9,10,11^ with a total of 569 patients were determined to satisfy our inclusion criteria with the appropriate report of outcomes of interest.

### Study Characteristics and Risk of Bias

Of the 7 included studies, 6^5,6,7,9,10,11^ analyzed data from prospective databases and 4^5,6,10,11^ were multicenter studies. Sample sizes of the included studies ranged from 21 to 185 patients. Six studies^5,6,8,9,10,11^ were determined to have good quality and 1 study^7^ was determined to have fair quality (eTable in Supplement 1). Patient characteristics, including age, sex, baseline National Institutes of Health Stroke Scale (NIHSS) or Alberta Stroke Program Early CT Score (ASPECTS) scores, and use of intravenous thrombolytics, are detailed in the Table.

**Table.** Characteristics of Patients in the Included Studies

Study	No. of patients	Age, mean (SD), y	NIHSS score, mean (SD)	Sex, No. (%)		Onset to puncture, median (IQR), h	IV thrombolysis, No. (%)	ASPECTS score, median (IQR)	sICH definition
				Men	Women				
Casetta et al,^7^ 2022	34	70.7 (12.3)	13 (8.5- 19)	19 (55.9)	15 (44.1)	28.5 (26.3-31.4)	NA	NA	ECASS II
Desai et al,^5^ 2018	21	65.2 (11.1)	18.2 (5.9)	8 (38.1)	13 (61.9)	48 (30-72)	NA	NA	ECASS III
Dhillon et al,^6^ 2023	104	NA	12.7 (7.4)	60 (57.7)	44 (42.3)	33.3 (7.4)	20 (19.2)	NA	ECASS II
Ha et al,^8^ 2023	61	65 (14)	10 (6)	43 (70.5)	18 (29.5)	80.8 (43.8-194.2)^a^	0	NA	Clinical deterioration or increase in NIHSS score ≥4
Purrucker et al,^9^ 2022	43	75.5 (10.1)	13 (8-21)	20 (46.5)	23 (53.5)	43.6 (26.8-71.3)	3 (7.0)	9 (7-10)	PH2
Sarraj et al,^10^ 2023	185	69 (60-80)	14 (8-20)	95 (52.5)	86 (47.5)	NA	NA	7 (6-9)	PH2
Shaban et al,^11^ 2023	121	67 (13.3)	14 (9)	67 (55.4)	54 (44.6)	29.4 (24-92.3)^a^	5 (4.1)	8 (2)^b^	PH2

Abbreviations: ASPECTS, Alberta Stroke Program Early CT Score; ECASS, European Cooperative Acute Stroke Study; IV, intravenous; NA, not applicable; NIHSS, National Institutes of Health Stroke Scale; PH2, parenchymal hemorrhage; sICH, symptomatic intracranial hemorrhage.

^a^ Values are presented as median (range).

^b^ Value is presented as mean (SD).

Of the 569 included patients, 312 (55.3% [95% CI, 50.9%-59.6%]) were men and 253 (44.7%) were women. A total of 35 patients received intravenous thrombolytics (4.9% [95% CI, 1.9%-12.2%]; eFigure 2 in Supplement 1). The mean age of included patients was 68.9 years (95% CI, 65.9-72.2). The mean baseline NIHSS score was 13.6 (95% CI, 11.9-15.5), and the mean ASPECTS score was 7.9 (95% CI, 7.2-8.7). The mean last known well and/or onset to puncture time was 46.2 hours (95% CI, 32.4-65.9 hours; eFigure 3 in Supplement 1).

### Outcomes of Interest

#### Functional Independence

**Figure 1.**
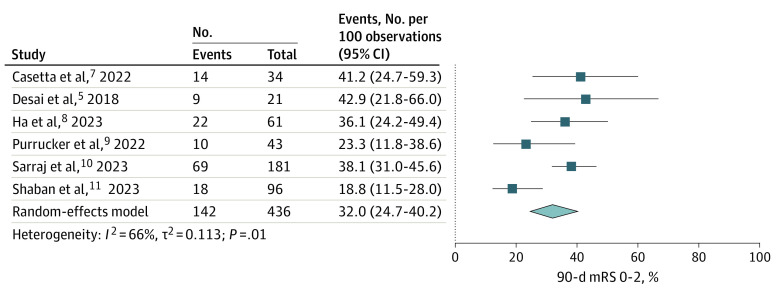
Frequencies of 90-day Modified Rankin Scale (mRS) Scores of 0 to 2

Six studies^5,7,8,9,10,11^ involving 463 patients reported on 90-day functional independence (the primary outcome) using mRS scores of 0 to 2, with a frequency of 32.0% (95% CI, 24.7%-40.2%). However, there was significant heterogeneity among the pooled studies (*I*^2^ = 66%, *P* = .01; Figure 1 and eFigure 4 in Supplement 1).

#### TICI Scores

**Figure 2.**
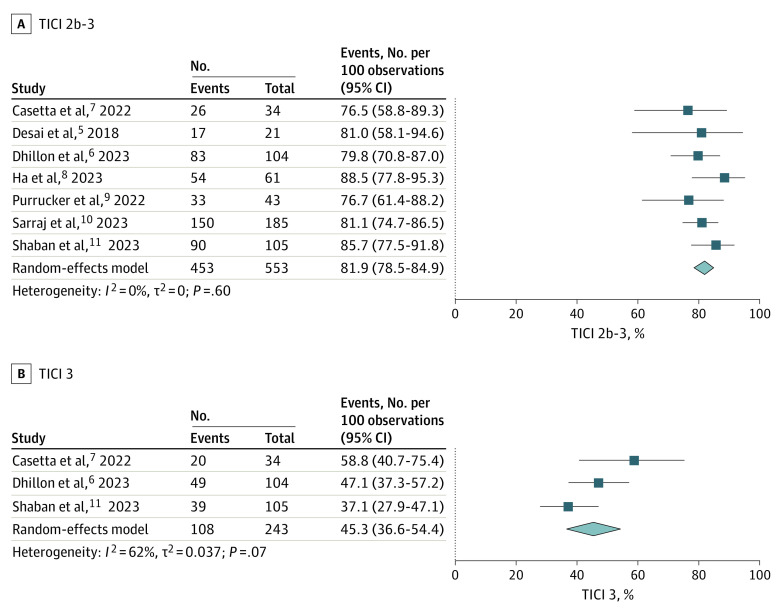
Frequencies of Thrombolysis in Cerebral Infarction Scores of 2b to 3 (TICI 2b-3) and 3 (TICI 3)

Seven studies^5,6,7,8,9,10,11^ involving 553 patients reported TICI scores of 2b to 3 and 3, with a frequency of 81.9% (95% CI, 78.5%-84.9%). There was no heterogeneity among the pooled studies (*I*^2^ = 0%, *P* = .60; Figure 2A and eFigure 5 in Supplement 1). Three studies^6,7,11^ involving 243 patients reported TICI scores of 3, with a frequency of 45.3% (95% CI, 36.6%-54.4%); however, there was moderate heterogeneity among the pooled studies (*I*^2^ = 62%, *P* = .07; Figure 2B and eFigure 6 in Supplement 1).

#### sICH and Mortality

**Figure 3.**
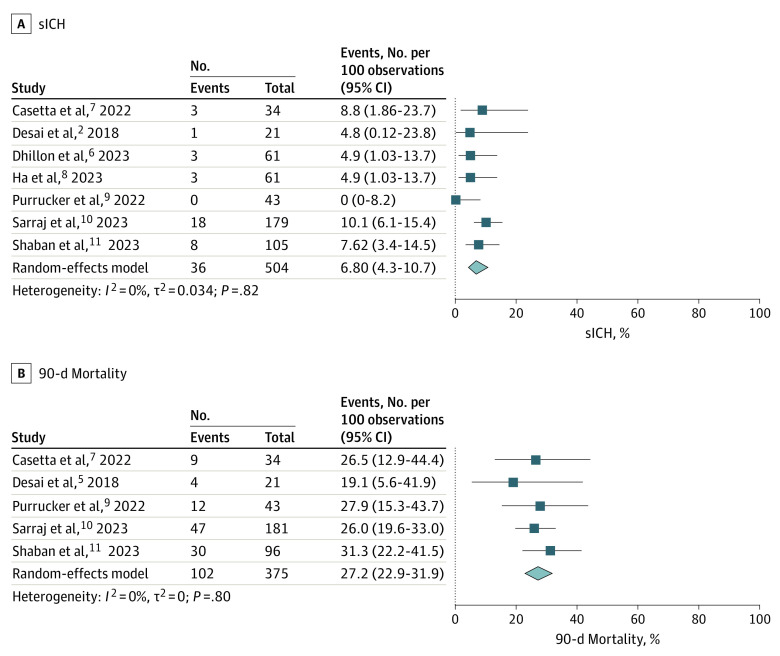
Frequencies of Symptomatic Intracranial Hemorrhage (sICH) and 90-day Mortality

Seven studies^5,6,7,8,9,10,11^ involving 504 patients reported on sICH, with a frequency of 6.8% (95% CI, 4.3%-10.7%; Figure 3A and eFigure 7 in Supplement 1). Five studies^5,7,9,10,11^ involving 375 patients reported on 90-day mortality, with a frequency of 27.2% (95% CI, 22.9%-31.9%; Figure 3B and eFigure 8 in Supplement 1). For both outcomes, no heterogeneity was detected among the included studies.

#### ENI and END

**Figure 4.**
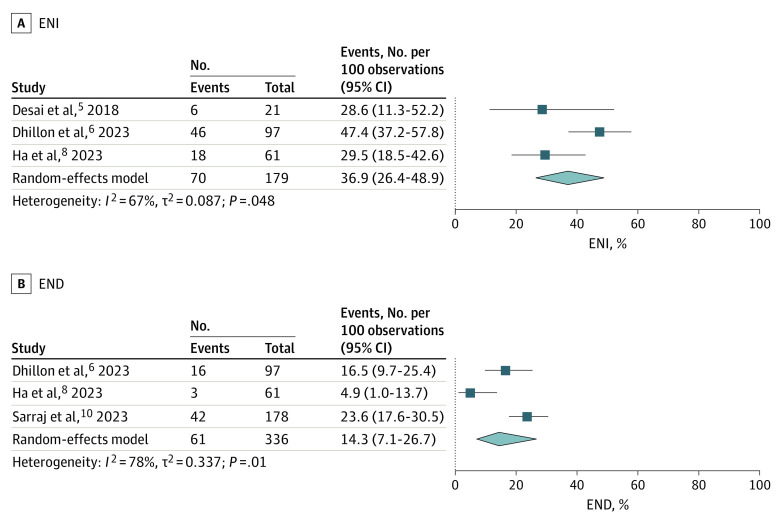
Frequencies of Early Neurological Improvement (ENI) and Early Neurological Deterioration (END)

Three studies^5,6,8^ involving 179 patients reported on ENI, with a frequency of 36.9% (95% CI, 26.4%-48.9%). However, there was significant heterogeneity among the pooled studies (*I*^2^ = 67%, *P* = .048; Figure 4A and eFigure 9 in Supplement 1). Three studies^6,8,10^ involving 336 patients reported on END, with a frequency of 14.3% (95% CI, 7.1%-26.7%). However, there was significant heterogeneity among the pooled studies (*I*^2^ = 78%, *P* = .01; Figure 4B and eFigure 10 in Supplement 1).

## Discussion

The findings of this systematic review and meta-analysis suggest that EVT may be safe and associated with favorable outcomes for AIS even if performed more than 24 hours since last known well. To our knowledge, the included studies represent the largest reported sample size (n = 569) of patients with stroke from LVO undergoing EVT within the very late window. Approximately one-third of patients (32.0%) treated with EVT in the very late window were functionally independent at 3 months despite having disabling deficits at the time of presentation. Successful reperfusion was achieved in 81.9% of patients and sICH was uncommon.

Determination of eligibility for EVT in the late and very late windows incorporates not only temporal considerations but also evidence of a relatively small ischemic core on imaging (computed tomography perfusion [CTP] or magnetic resonance imaging [MRI]–diffusion-weighted imaging [DWI]^2,3^) and a salvageable penumbra either documented on imaging (CTP or DWI^2^) or assumed based on clinical symptom severity.^3^ The studies included in our present analysis used noncontrast CT, CT angiography, CTP imaging, and MRI-DWI to select patients for EVT. It is yet to be determined which imaging modality is preferred in this time window, although most studies included in our meta-analysis used advanced imaging. Selection bias is unavoidable in these types of nonrandomized studies exploring the value of an intervention among patients who do not meet current criteria for its indication. Nonetheless, the fact that very late use of EVT can result in roughly one-third of patients being functionally independent at 3 months is remarkable, even if those patients were carefully selected. Although there may be utility for EVT in the very late window, these results should not inform less aggressive treatment in earlier time points, where superiority of EVT has been proven through RCTs over medical therapy. Evidence for expanded thrombectomy indications continues to accumulate, as shown by the recent large core RCTs; our current study points to the need for an RCT with patients presenting beyond 24 hours.^17,18^

To our knowledge, no previous meta-analyses have been published regarding patients with very late-window AIS undergoing EVT. The AURORA (Analysis of Pooled Data From Randomized Studies of Thrombectomy More Than 6 Hours After Last Known Well) study investigators meta-analyzed outcomes following EVT for late-window AIS (6-24 hours) at the individual patient level.^4^ Their meta-analysis included only RCTs and found that the frequency of 90-day functional independence in patients with late-window stroke undergoing EVT was 45.9%. In our study of patients treated in the very late window (>24 hours), the frequency of 90-day functional independence was 32.0%. While the frequency of functional independence reported here is lower than that reported in the AURORA study, it is very encouraging. Multiple factors may explain the lower frequency of 90-day functional independence observed in our study. First, it is reasonable to expect that patients treated in the very late window will achieve a lower frequency of functional independence than patients treated in the late window because shorter time from last known well to puncture time is associated with a higher frequency of reperfusion and better outcomes.^19^ Second, the AURORA meta-analysis represented results from RCTs only. Our study analyzed clinical data, which differ substantially from RCTs. A previous study found that only one-third of patients in a clinical cohort met the stringent enrollment criteria of RCTs.^20^ Furthermore, a sizeable number of patients included in our study had posterior circulation AIS.^9,11^ The RCTs for late-window stroke published to date have focused only on AC-LVO, and it has been previously reported that patients with posterior circulation AIS experience worse outcomes than those with anterior circulation AIS.^21^ Lastly, to put our findings in context, it is useful to recognize that the frequency of 90-day functional independence we observed in this meta-analysis of patients treated more than 24 hours after last known well time was actually comparable to that observed in the seminal MR CLEAN trial (Multicenter Randomized Clinical Trial of Endovascular Treatment for Acute Ischemic Stroke in the Netherlands) among patients treated with EVT within 6 hours from symptom onset (90-day functional independence was 32.6%).^22^

The safety outcomes reported in our meta-analysis compare favorably with those of the AURORA meta-analysis.^4^ The frequency of 90-day mortality in the AURORA study was 16.5%, while it was 27.2% in our study. Furthermore, the frequency of sICH in the AURORA study was 5.3%, while it was 6.8% in our study. These results suggest that EVT in the very late window is associated with low rates of complications and may potentially be associated with a similar safety profile to that of the late window. This is encouraging, since the safety profile of very late-window EVT should therefore allow for future studies to be performed in this patient population. Additionally, the frequencies of TICI scores 2b to 3 (81.9%) and 3 (45.3%) in our analysis suggest that very late-window EVT may be an effective procedure with favorable technical success frequencies. Although EVT was associated with favorable technical success rates across included studies, outcomes were not stratified by technique used. The studies included in our meta-analysis generally used aspiration thrombectomy, stent retrievers, or combination treatment. Unsurprisingly, the frequency of intravenous thrombolysis was low among included patients.

### Limitations

Our study has limitations. First, a low number of studies met our inclusion criteria, and certain outcomes were not reported in all studies. This is expected, as very late-window AIS from LVO is a relatively uncommon presentation, and very late-window AIS undergoing EVT is even less common. Second, our meta-analysis did not have a comparison group treated with medical management, as many of the included studies did not include a comparison group. Third, we did not have access to patient-level data, which limited the analysis we were able to perform. Furthermore, ENI and END have been defined loosely in the literature. Finally, stroke onset was reported as witnessed, unwitnessed, or wake-up stroke. Because studies did not stratify patients based on stroke onset, we could not account for potential differences. Future RCTs and prospective, comparative studies are needed to determine the safety and efficacy of EVT in the very late window, and a patient-level meta-analysis should be performed with a larger sample size to determine which patients may benefit most from the endovascular intervention.

## Conclusions

In this meta-analysis of clinical data from 569 patients undergoing EVT for AIS from LVO in the very late window (>24 hours from last known well), we observed that the overall frequency of 90-day functional independence was 32.0%. Furthermore, we observed that EVT was associated with favorable frequencies of TICI scores of 2b to 3 and 3 and a relatively low frequency of 90-day mortality and sICH. Future RCTs and prospective, comparative studies and subsequent patient-level meta-analysis should be conducted to further investigate the use of EVT in the very late window.

## Notes

Supplement 1. **eFigure 1.** PRISMA Diagram Detailing the Literature Search Process **eFigure 2.** Forest Plot of Frequencies of Male Sex and IV tPA **eFigure 3.** Forest Plot of Age, Baseline NIHSS, ASPECTS, and Onset to Puncture Time **eFigure 4.** Funnel Plot for 90-day mRS 0-2 **eFigure 5.** Funnel Plot for TICI 2b-3 **eFigure 6.** Funnel Plot for TICI 3 **eFigure 7.** Funnel Plot for sICH **eFigure 8.** Funnel Plot for 90-day Mortality **eFigure 9.** Funnel Plot for ENI **eFigure 10.** Funnel Plot for END **eAppendix.** Search Strategy **eTable.** Detailed Risk-of-Bias Assessment

Supplement 2.
Data Sharing Statement

## References

[1] Goyal M, Menon BK, van Zwam WH, ; HERMES Collaborators. Endovascular thrombectomy after large-vessel ischaemic stroke: a meta-analysis of individual patient data from five randomised trials. Lancet. 2016;387(10029):1723-1731. doi:10.1016/S0140-6736(16)00163-X 26898852

[2] Albers GW, Marks MP, Kemp S, ; DEFUSE 3 Investigators. Thrombectomy for stroke at 6 to 16 hours with selection by perfusion imaging. N Engl J Med. 2018;378(8):708-718. doi:10.1056/NEJMoa1713973 29364767 PMC6590673

[3] Nogueira RG, Jadhav AP, Haussen DC, ; DAWN Trial Investigators. Thrombectomy 6 to 24 hours after stroke with a mismatch between deficit and infarct. N Engl J Med. 2018;378(1):11-21. doi:10.1056/NEJMoa1706442 29129157

[4] Jovin TG, Nogueira RG, Lansberg MG, . Thrombectomy for anterior circulation stroke beyond 6 h from time last known well (AURORA): a systematic review and individual patient data meta-analysis. Lancet. 2022;399(10321):249-258. doi:10.1016/S0140-6736(21)01341-6 34774198

[5] Desai SM, Haussen DC, Aghaebrahim A, . Thrombectomy 24 hours after stroke: beyond DAWN. J Neurointerv Surg. 2018;10(11):1039-1042. doi:10.1136/neurintsurg-2018-013923 29807887

[6] Dhillon PS, Butt W, Podlasek A, . Endovascular thrombectomy beyond 24 hours from ischemic stroke onset: a propensity score matched cohort study. J Neurointerv Surg. 2023;15(3):233-237. 35169031 10.1136/neurintsurg-2021-018591

[7] Casetta I, Fainardi E, Pracucci G, ; Italian Registry of Endovascular Thrombectomy in Acute Stroke (IRETAS). Endovascular treatment beyond 24 hours from the onset of acute ischemic stroke: the Italian Registry of Endovascular Thrombectomy in Acute Stroke (IRETAS). J Neurointerv Surg. 2022;14(12):1186-1188. 34732532 10.1136/neurintsurg-2021-018045

[8] Ha SH, Ryu JC, Bae JH, . Early response to endovascular thrombectomy after stroke: early, late, and very late time windows. Cerebrovasc Dis. 2023;52(1):28-35. doi:10.1159/000525083 35671740

[9] Purrucker JC, Ringleb PA, Seker F, . Leaving the day behind: endovascular therapy beyond 24 h in acute stroke of the anterior and posterior circulation. Ther Adv Neurol Disord. Published online May 25, 2022. doi:10.1177/17562864221101083 35646160 PMC9136439

[10] Sarraj A, Kleinig TJ, Hassan AE, . Association of endovascular thrombectomy vs medical management with functional and safety outcomes in patients treated beyond 24 hours of last known well: the SELECT Late study. JAMA Neurol. 2023;80(2):172-182. doi:10.1001/jamaneurol.2022.4714 36574257 PMC9857518

[11] Shaban A, Al Kasab S, Chalhoub RM, . Mechanical thrombectomy for large vessel occlusion strokes beyond 24 hours. J Neurointerv Surg. Published online January 2, 2023. doi:10.1136/jnis-2022-019372 36593118

[12] Higgins JPT, Thomas J, Chandler J, , eds. Cochrane Handbook for Systematic Reviews of Interventions, Version 6.3. Cochrane Collaboration; 2022.

[13] Lo CK, Mertz D, Loeb M. Newcastle-Ottawa Scale: comparing reviewers’ to authors’ assessments. BMC Med Res Methodol. 2014;14:45. doi:10.1186/1471-2288-14-45 24690082 PMC4021422

[14] Lin L, Xu C, Chu H. Empirical comparisons of 12 meta-analysis methods for synthesizing proportions of binary outcomes. J Gen Intern Med. 2022;37(2):308-317. doi:10.1007/s11606-021-07098-5 34505983 PMC8432281

[15] Higgins JP, Green S. Cochrane Handbook for Systematic Reviews of Interventions (Identifying and Measuring Heterogeneity), Version 5.1.0. Cochrane Collaboration; 2011.

[16] Ghozy S, Nam NH, Radwan I, . Therapeutic efficacy of hepatitis B virus vaccine in treatment of chronic HBV infections: a systematic review and meta-analysis. Rev Med Virol. 2020;30(3):e2089. doi:10.1002/rmv.2089 31811678

[17] Sarraj A, Hassan AE, Abraham MG, ; SELECT2 Investigators. Trial of endovascular thrombectomy for large ischemic strokes. N Engl J Med. 2023;388(14):1259-1271. doi:10.1056/NEJMoa2214403 36762865

[18] Huo X, Ma G, Tong X, ; ANGEL-ASPECT Investigators. Trial of endovascular therapy for acute ischemic stroke with large infarct. N Engl J Med. Published online February 10, 2023. doi:10.1056/NEJMoa2213379 36762852

[19] Bourcier R, Goyal M, Liebeskind DS, ; HERMES Trialists Collaboration. Association of time from stroke onset to groin puncture with quality of reperfusion after mechanical thrombectomy: a meta-analysis of individual patient data from 7 randomized clinical trials. JAMA Neurol. 2019;76(4):405-411. doi:10.1001/jamaneurol.2018.4510 30667465 PMC6459219

[20] Deb-Chatterji M, Pinnschmidt H, Flottmann F, . Stroke patients treated by thrombectomy in real life differ from cohorts of the clinical trials: a prospective observational study. BMC Neurol. 2020;20(1):81. doi:10.1186/s12883-020-01653-z 32138684 PMC7059360

[21] Adusumilli G, Pederson JM, Hardy N, . Mechanical thrombectomy in anterior vs. posterior circulation stroke: a systematic review and meta-analysis. Interv Neuroradiol. Published online May 13, 2022. doi:10.1177/15910199221100796 35549748 PMC11310733

[22] Berkhemer OA, Fransen PSS, Beumer D, ; MR CLEAN Investigators. A randomized trial of intraarterial treatment for acute ischemic stroke. N Engl J Med. 2015;372(1):11-20. doi:10.1056/NEJMoa1411587 25517348

