# Supplementary material for: Endovascular Therapy for Stroke Presenting Beyond 24 Hours: A Systematic Review and Meta-analysis
Source: JAMA Netw Open. 2023 May 4;6(5):e2311768. doi: 10.1001/jamanetworkopen.2023.11768 (PMC10160871; doi:10.1001/jamanetworkopen.2023.11768)
Supplement: Supplement 1. — eFigure 1. PRISMA Diagram Detailing the Literature Search Process eFigure 2. Forest Plot of Frequencies of Male Sex and IV tPA eFigure 3. Forest Plot of Age, Baseline NIHSS, ASPECTS, and Onset to Puncture Time eFigure 4. Funnel Plot for 90-day mRS 0-2 eFigure 5. Funnel Plot for TICI 2b-3 eFigure 6. Funnel Plot for TICI 3 eFigure 7. Funnel Plot for sICH eFigure 8. Funnel Plot for 90-day Mortality eFigure 9. Funnel Plot for ENI eFigure 10. Funnel Plot for END eAppendix. Search Strategy eTable. Detailed Risk-of-Bias Assessment [file jamanetwopen-e2311768-s001.pdf]

Supplementary Online Content

Kobeissi H, Ghozy S, Adusumilli G, et al. Endovascular therapy for stroke presenting beyond 24 hours: a systematic review and meta-analysis. *JAMA Netw Open*. 2023;6(5):e2311768. doi:10.1001/jamanetworkopen.2023.11768

eFigure 1. PRISMA Diagram Detailing the Literature Search Process

eFigure 2. Forest Plot of Frequencies of Male Sex and IV tPA

eFigure 3. Forest Plot of Age, Baseline NIHSS, ASPECTS, and Onset to Puncture Time

eFigure 4. Funnel Plot for 90-day mRS 0-2

eFigure 5. Funnel Plot for TICI 2b-3

eFigure 6. Funnel Plot for TICI 3

eFigure 7. Funnel Plot for sICH

eFigure 8. Funnel Plot for 90-day Mortality

eFigure 9. Funnel Plot for ENI

eFigure 10. Funnel Plot for END

eAppendix. Search Strategy

eTable. Detailed Risk-of-Bias Assessment

This supplementary material has been provided by the authors to give readers additional information about their work.

© 2023 Kobeissi H et al. *JAMA Network Open*.

eFigure 1. PRISMA Diagram Detailing the Literature Search Process

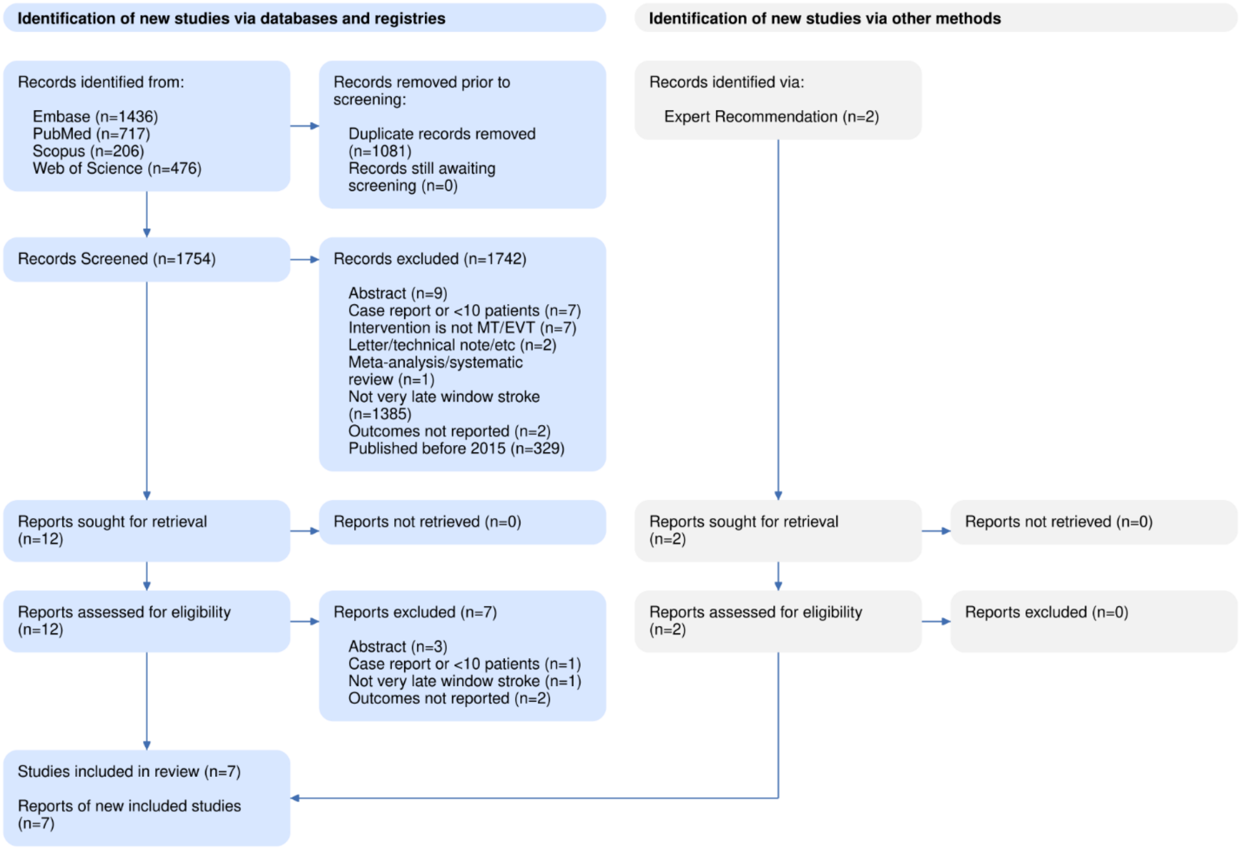

eFigure 2. Forest Plot of Frequencies of Male Sex and IV tPA

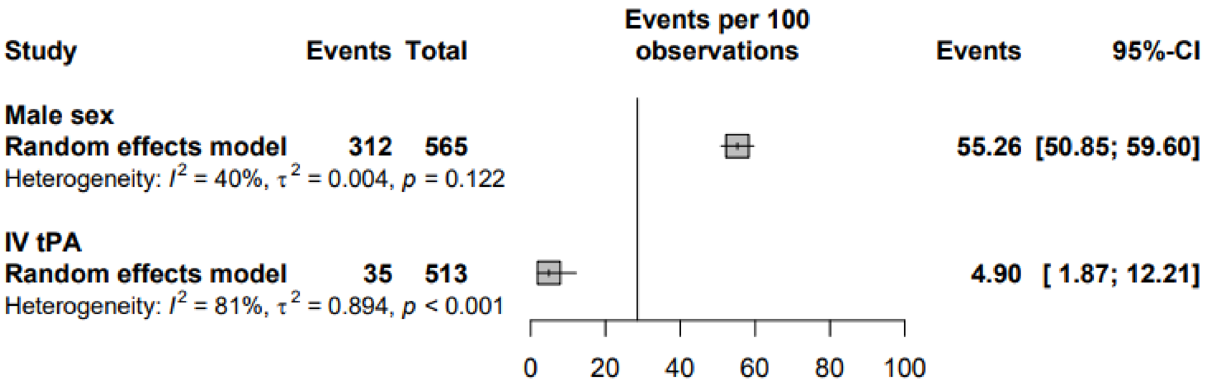

© 2023 Kobeissi H et al. *JAMA Network Open*.

eFigure 3. Forest Plot of Age, Baseline NIHSS, ASPECTS, and Onset to Puncture Time

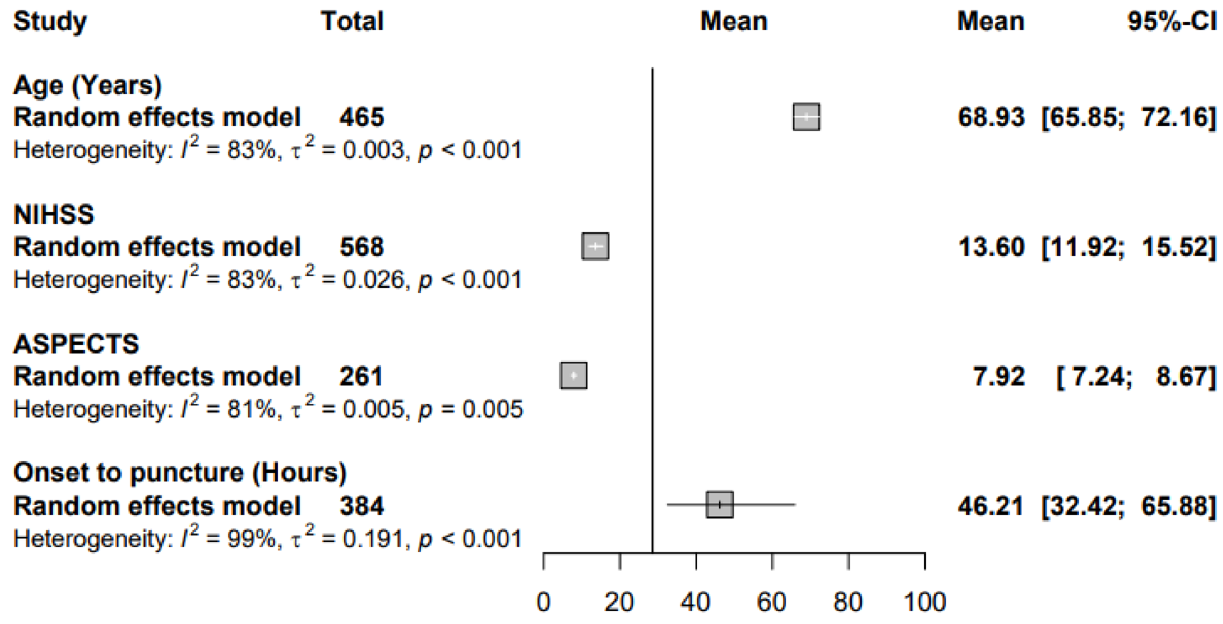

eFigure 4. Funnel Plot for 90-day mRS 0-2

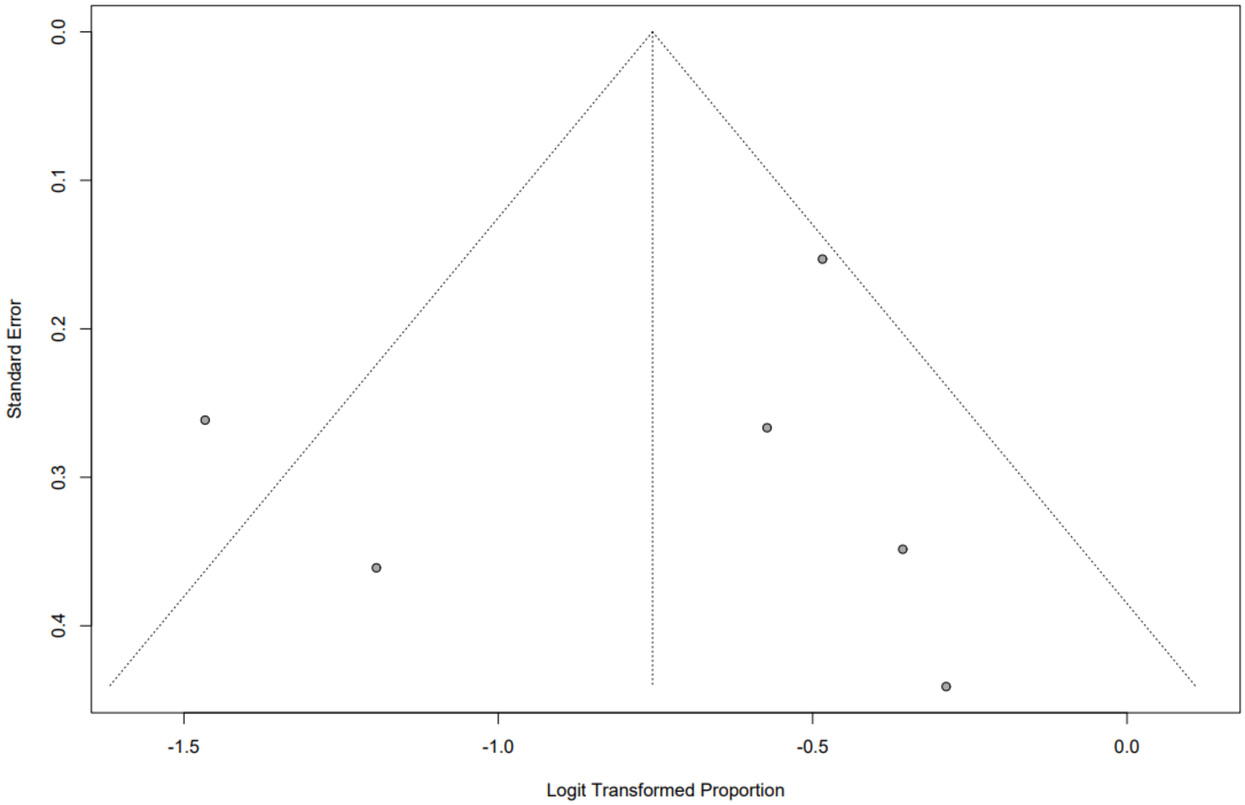

© 2023 Kobeissi H et al. *JAMA Network Open*.

eFigure 5. Funnel Plot for TICI 2b-3

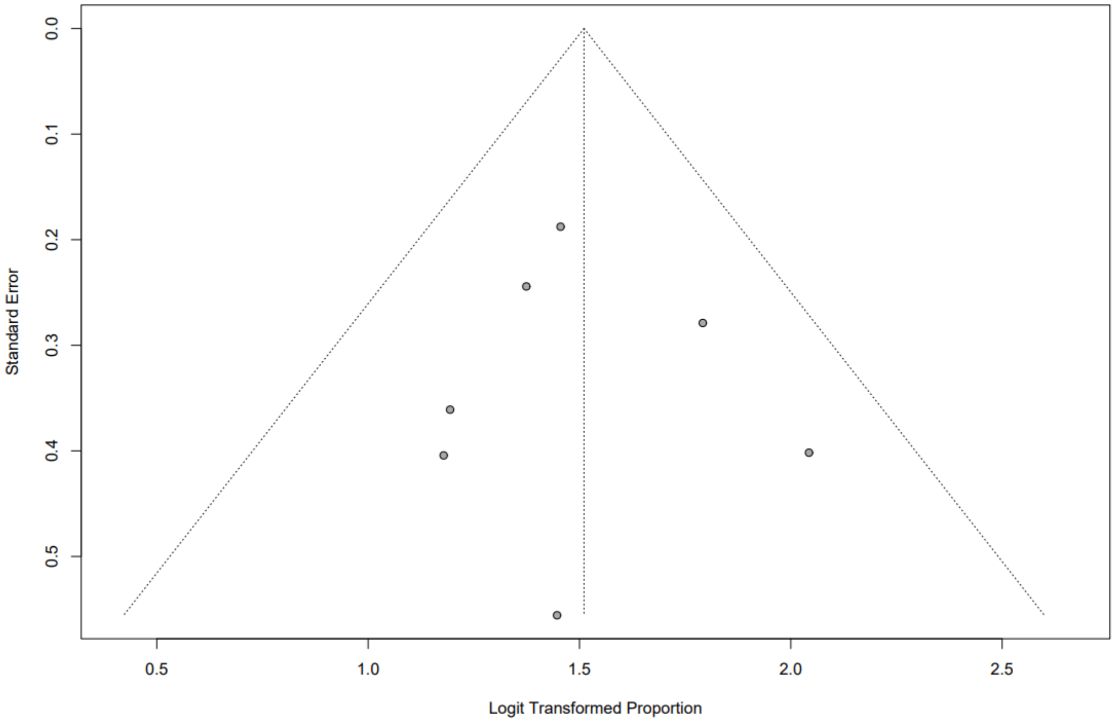

eFigure 6. Funnel Plot for TICI 3

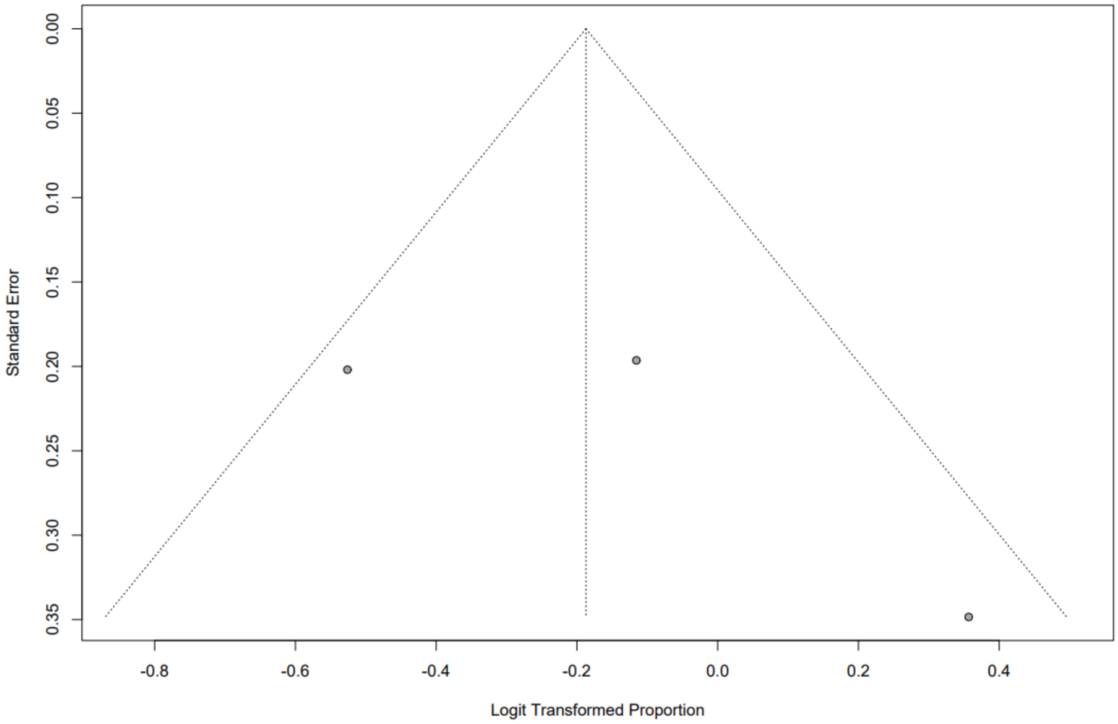

© 2023 Kobeissi H et al. *JAMA Network Open*.

eFigure 7. Funnel Plot for sICH

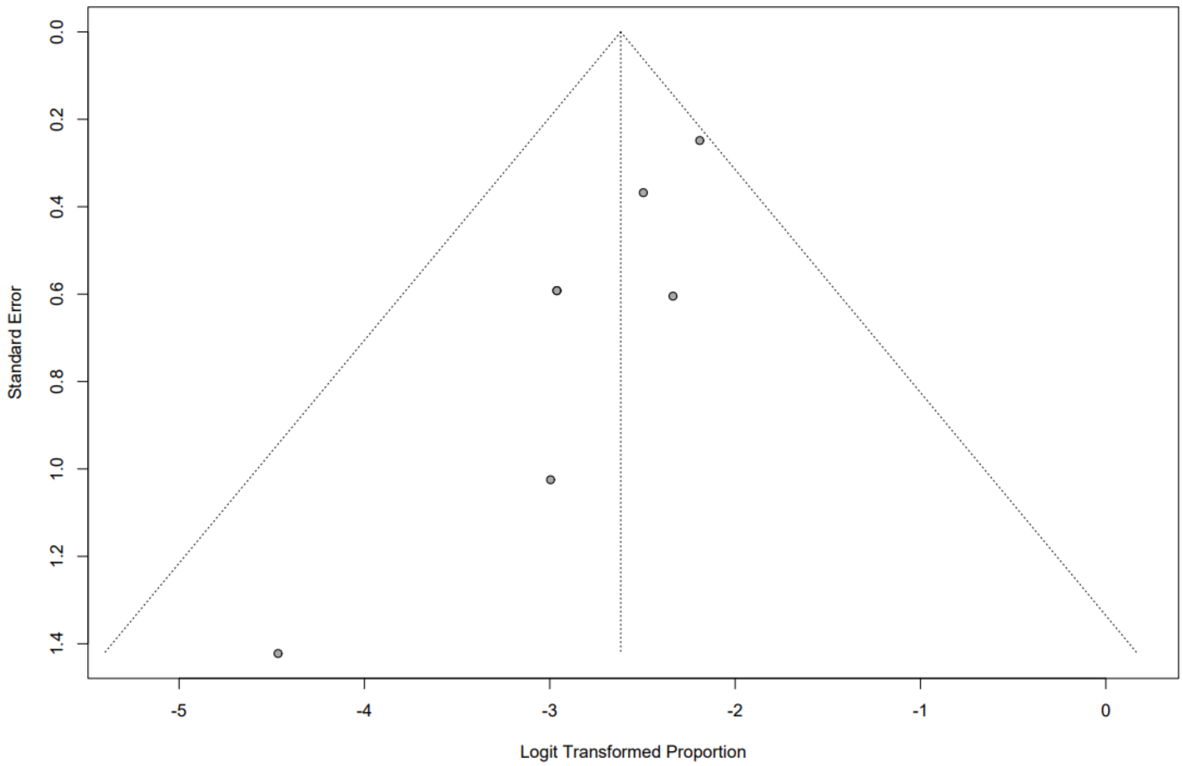

eFigure 8. Funnel Plot for 90-day Mortality

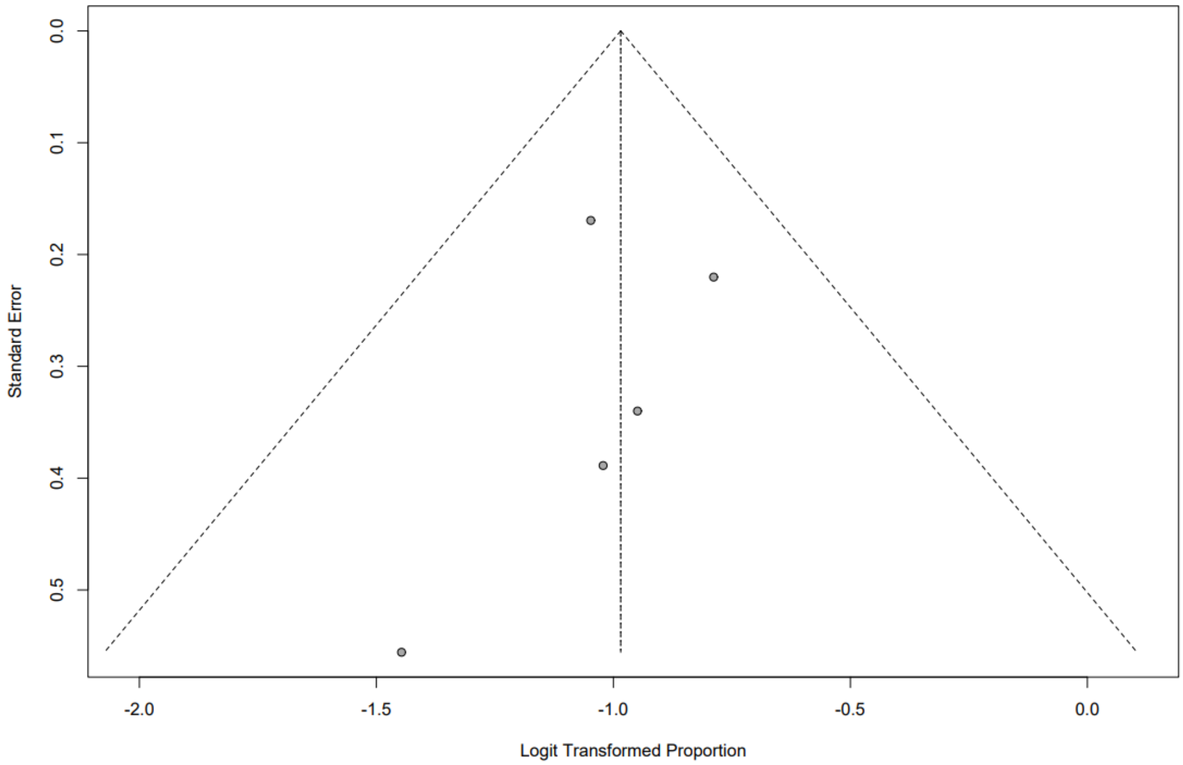

© 2023 Kobeissi H et al. *JAMA Network Open*.

eFigure 9. Funnel Plot for ENI

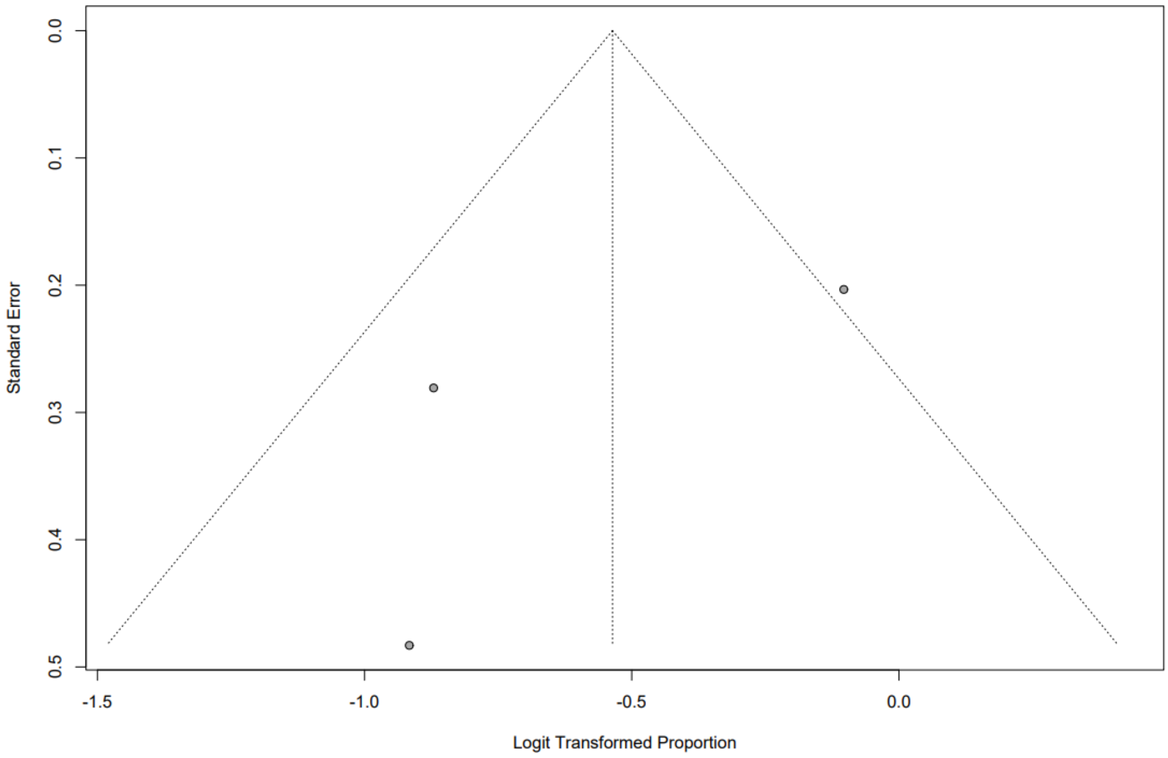

eFigure 10. Funnel Plot for END

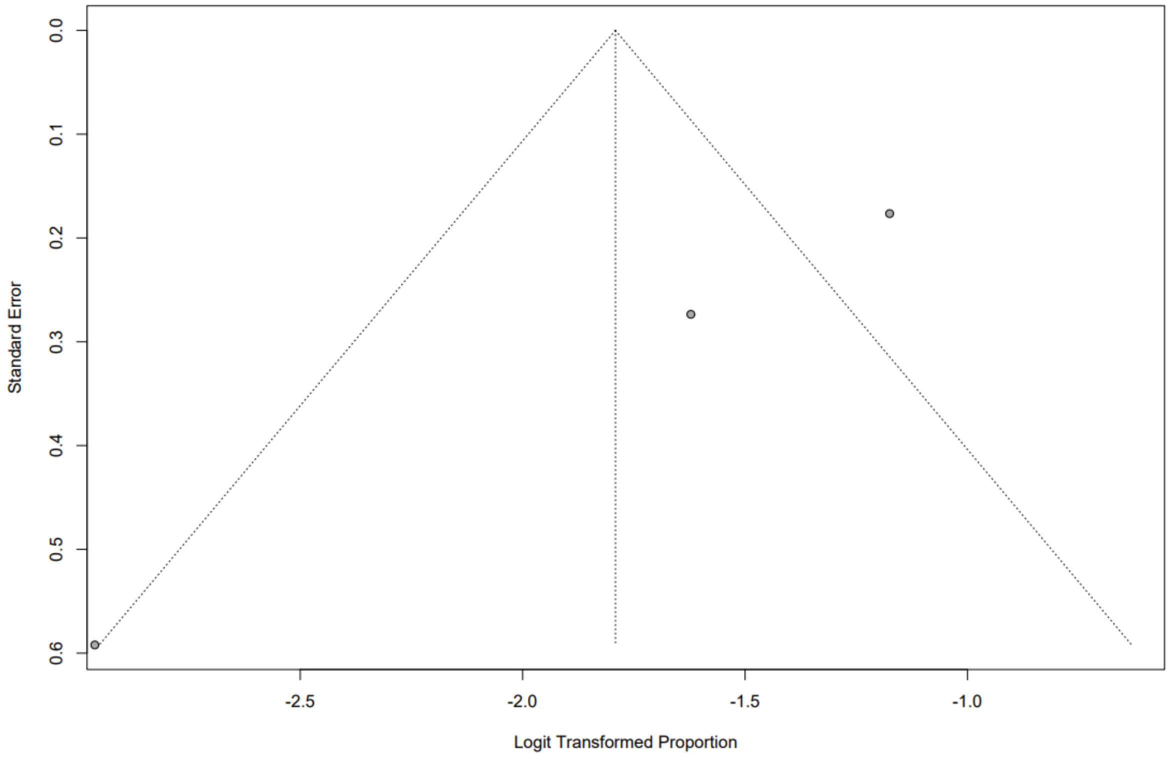

© 2023 Kobeissi H et al. *JAMA Network Open*.

eAppendix. Search Strategy

Search	Database	Query	Date	Results	Duplicate	Excluded	Included
1	PubMed	(("Stroke"[Title]) AND ("Thrombectomy"[Title] OR "endovascular"[Title]) AND ("late"[Title/Abstract] OR "very late"[Title/Abstract] OR "beyond 24 hours"[Title/Abstract] OR "beyond 24h"[Title/Abstract] OR "beyond 24 h"[Title/Abstract] OR ">24"[Title/Abstract])) NOT ("Systematic Review"[Publication Type] OR "Review"[Publication Type] OR "Case Reports"[Publication Type])	Dec 13, 2022	717	0	711	5
2	Embase	Embase <1974 to 2022 December 12> 1 "Stroke".ti. 191105 2 "Thrombectomy".ti. 10146 3 "endovascular".ti. 41176 4 2 or 3 50025 5 ("late" or "very late" or "beyond 24 hours" or "beyond 24h" or "beyond 24 h" or ">24").ab,ti. 2505634 6 1 and 4 and 5 1468	Dec 13, 2022	1436	557	879	0
3	Web of Science	# Web of Science Search Strategy (v0.1) # Database: Web of Science Core Collection # Entitlements: - WOS.SCI: 1975 to 2022 - WOS.ESCI: 2017 to 2022 # Searches: 1: TI=("stroke") Date Run: Tue Dec 13 2022 17:28:16 GMT-0600 (Central Standard Time) Results: 165403 2: TI=("Thrombectomy") Date Run: Tue Dec 13 2022 17:28:33 GMT-0600 (Central Standard Time) Results: 7881 3: TS=("late" OR "very late" OR "beyond 24 hours" OR "beyond 24h"	Dec 13, 2022	476	445	31	0

© 2023 Kobeissi H et al. *JAMA Network Open*.

		OR "beyond 24 h" OR ">24") Date Run: Tue Dec 13 2022 17:29:16 GMT-0600 (Central Standard Time) Results: 2194858 4: #3 AND #2 AND #1 Date Run: Tue Dec 13 2022 17:29:25 GMT-0600 (Central Standard Time) Results: 477					
4	Scopus	(TITLE-ABS("Stroke")) AND (TITLE-ABS("Thrombectomy")) AND (TITLE-ABS("late" OR "very late" OR "beyond 24 hours" OR "beyond 24h" OR "beyond 24 h" OR ">24"))	Dec 13, 2022	206	79	127	0
5	Expert Recommendation		Dec 28, 2022	2	0	0	2
TOTAL				2837	1081	1749	7

© 2023 Kobeissi H et al. *JAMA Network Open*.

eTable. Detailed Risk-of-Bias Assessment

				New Castle Ottawa scale assessment (NOS)								
				Selection				Comparability	Outcome			
	Study	Year	Sample (n)	Representativeness of the exposed cohort	Selection of the non exposed cohort	Ascertainment of exposure	Demonstration that outcome of interest was not present at start of study	Comparability of cohorts on the basis of the design or analysis	Assessment of outcome	Was follow-up long enough for outcomes to occur	Adequacy of follow up of cohorts	Quality Score
1	Desai	2018	21	*	*	*		*	*	*	*	Good
2	Shaban	2023	121	*	*	*		*	*	*	*	Good
3	Dhillon	2022	104	*	*	*		*	*	*	*	Good
4	Casetta	2022	34		*	*		*	*	*	*	Fair
5	Ha	2022	61	*	*	*		*	*	*	*	Good
6	Purrucker	2022	45	*	*	*		*	*	*	*	Good
7	Sarraj	2022	185	*	*	*		*	*	*	*	Good

© 2023 Kobeissi H et al. *JAMA Network Open*.
