# Supplementary material for: Endovascular Therapy for Stroke Presenting Beyond 24 Hours: A Systematic Review and Meta-analysis
Source: JAMA Netw Open. 2023 May 4;6(5):e2311768. doi: 10.1001/jamanetworkopen.2023.11768 (PMC10160871; doi:10.1001/jamanetworkopen.2023.11768)
Supplement: Supplement 2. — Data Sharing Statement [file jamanetwopen-e2311768-s002.pdf]

Data Sharing Statement

Kobeissi. Endovascular Therapy for Stroke Presenting Beyond 24 Hours. *JAMA Netw Open*. Published May 04, 2023. doi:10.1001/jamanetworkopen.2023.11768

Data
Data available: No

Additional Information
Explanation for why data not available: The data that support this study comes from publicly available papers, which are cited in our systematic review and meta-analysis.
